# The holobiont transcriptome of teneral tsetse fly species of varying vector competence

**DOI:** 10.1186/s12864-021-07729-5

**Published:** 2021-05-31

**Authors:** Miguel Medina Munoz, Caitlyn Brenner, Dylan Richmond, Noah Spencer, Rita V. M. Rio

**Affiliations:** 1 Department of Biology, Eberly College of Arts and Sciences, West Virginia University, Morgantown, WV 26505 USA; 2 Department of Biology, Washington and Jefferson College, Washington, PA 15301 USA

**Keywords:** Symbiosis, RNA-Seq, *Wigglesworthia*, *Sodalis*, Tsetse, Teneral, *Glossina*, Vector competence

## Abstract

**Background:**

Tsetse flies are the obligate vectors of African trypanosomes, which cause Human and Animal African Trypanosomiasis. Teneral flies (newly eclosed adults) are especially susceptible to parasite establishment and development, yet our understanding of why remains fragmentary. The tsetse gut microbiome is dominated by two Gammaproteobacteria, an essential and ancient mutualist *Wigglesworthia glossinidia* and a commensal *Sodalis glossinidius*. Here, we characterize and compare the metatranscriptome of teneral *Glossina morsitans* to that of *G. brevipalpis* and describe unique immunological, physiological, and metabolic landscapes that may impact vector competence differences between these two species.

**Results:**

An active expression profile was observed for *Wigglesworthia* immediately following host adult metamorphosis. Specifically, ‘translation, ribosomal structure and biogenesis’ followed by ‘coenzyme transport and metabolism’ were the most enriched clusters of orthologous genes (COGs), highlighting the importance of nutrient transport and metabolism even following host species diversification. Despite the significantly smaller *Wigglesworthia* genome more differentially expressed genes (DEGs) were identified between interspecific isolates (*n* = 326, ~ 55% of protein coding genes) than between the corresponding *Sodalis* isolates (*n* = 235, ~ 5% of protein coding genes) likely reflecting distinctions in host co-evolution and adaptation. DEGs between *Sodalis* isolates included genes involved in chitin degradation that may contribute towards trypanosome susceptibility by compromising the immunological protection provided by the peritrophic matrix. Lastly, *G. brevipalpis* tenerals demonstrate a more immunologically robust background with significant upregulation of IMD and melanization pathways.

**Conclusions:**

These transcriptomic differences may collectively contribute to vector competence differences between tsetse species and offers translational relevance towards the design of novel vector control strategies.

**Supplementary Information:**

The online version contains supplementary material available at 10.1186/s12864-021-07729-5.

## Background

Tsetse flies (Diptera: Glossinidae) inhabit much of sub-Saharan Africa in an area referred to as the “tsetse belt”, where significant detriment towards public health and agricultural sustainability occur due to the presence of these vectors [1, 2]. Tsetse adults of both sexes are strictly hematophagous, and thus, have epidemiological significance as the cyclical (and obligate) vectors of human and animal African trypanosomes. Because there are no vaccines and few pharmaceuticals available, vector control [3–5] remains a significant component of programs intended to impede the transmission of trypanosome infections.

Tsetse flies have a viviparous reproductive biology [6] characterized by the ‘in utero’ development of a single larva during each gonotrophic cycle. Here, the larva receives nutritional lipids and proteins through maternal secretions from modified female accessory reproductive glands known as milk glands. These milk gland secretions also seed progeny with the core bacteria of the tsetse digestive tract microbiota [7–9], specifically the obligate mutualist *Wigglesworthia glossinidia* [10] and the commensal *Sodalis glossinidius* [11] (hereafter *Wigglesworthia* and *Sodalis*, respectively). Although a more complex microbial diversity in the digestive tracts of adult flies has been reported, these environmentally acquired bacteria are lacking within tenerals (newly emerged adults which have not yet fed), are transient, and not integrated into tsetse biology due to their random occurrence [12–14].

Despite sharing a common route of maternal inheritance, the *Wigglesworthia* and *Sodalis* endosymbionts have distinct coevolutionary histories with their tsetse host. The [14] ancient mutualist, *Wigglesworthia*, likely established at the commencement (or soon thereafter) of tsetse species radiation with subsequent co-diversification across host species spanning the course of 50–80 million years [15]. The extraordinary significance of the *Wigglesworthia* mutualism towards tsetse biology is symbolized by the bacteriome structure [16], which exclusively harbors *Wigglesworthia* intracellularly within specialized tsetse epithelial cells (known as bacteriocytes) in an immunotolerant niche [17] in return for nutrient supplementation of the limited blood diet. In contrast, *Sodalis* symbionts have a wide tropism being found in the tsetse digestive tract, muscle, hemolymph, salivary glands and fat body [9, 18]. Unlike *Wigglesworthia*, *Sodalis* has established much more recently as reflected by its stochastic distribution in wild tsetse populations [19–23] which also indicates that this symbiosis is not a requisite for tsetse survival although its role towards tsetse vector competence remains contentious [24].

Age impacts tsetse vector competence as teneral flies have higher permissiveness towards trypanosome infections upon acquisition of their first blood meal (referred to as the “teneral phenomenon” reviewed in [25]). Although numerous microbiota contributions towards tsetse biology have been functionally characterized [26–28], the contributions of *Wigglesworthia* and *Sodalis* to the “teneral phenomenon” remain poorly understood. Here, we characterize and compare the *Wigglesworthia*, *Sodalis* and tsetse transcriptomes within teneral flies of low (*Glossina brevipalpis*) versus high (*G. morsitans*) vector competence. We present differences in the gene expression profiles of tsetse, *Wigglesworthia* and *Sodalis* that likely contribute towards unique immunological, physiological and metabolic landscapes that may impact vector competence. In particular, we focus on genes involved in nutrient provisioning with *Wigglesworthia*, genes that may disrupt tsetse physiology and facilitate trypanosome infections with *Sodalis*, and immunity in teneral tsetse of different species. These findings have significance not only towards understanding the basic biology of tsetse and its microbiota during the incipient stages of adulthood, but also applied merit towards identifying biological factors which may predispose certain tsetse species towards higher trypanosome infection rates which can be used in the design of novel control mechanisms.

## Results

### General transcriptome features

Eighteen cDNA libraries were generated from the total homogenates of either sex-specific bacteriomes or midguts from *G. brevipalpis* and *G. morsitans* teneral (1 day old, unfed virgin) tsetse flies. For each species, we had a collection of 6 bacteriome libraries and 3 midgut libraries. Each library consisted of a pool of either 20 bacteriomes or midguts. The libraries were sequenced using Illumina HiSeq technology, resulting in an average of 17,390,200 ± 2,558,126 (Std. dev) of paired-end reads (2 × 51 bp in length) from each sample. All reads were scored as “very good” quality (Phred scores > 30, Additional file 1: Figure S1). Reads were mapped to a reference dataset consisting of the collective genomes of tsetse species (*G. brevipalpis* and *G. morsitans*), *Wigglesworthia* isolates (*W. glossinidia morsitans* and *W. glossinidia brevipalpis*), and *S. glossinidius* (Additional file 1: Figure S2) using Salmon [29]. Importantly, the majority of reads mapped back only to their corresponding genomes serving as a control for specificity. A total of 112,895,620 reads (corresponding to ~ 63.2% of mapped reads) were identified as having tsetse origin, 65,550,668 reads (corresponding to ~ 36.7% of mapped reads) mapped to *Wigglesworthia* and 214,292 reads (corresponding to ~ 0.1% of mapped reads) were recognized as *Sodalis*. The lower abundance of *Sodalis* reads relative to *Wigglesworthia* likely arises due to a significantly lower population density at the teneral stage [30]. Non-specific reads (i.e. reads that mapped back to the genomes which were not of origin) were excluded from further analyses. There were no significant differences in either the mean abundance of total reads (total *G. brevipalpis* mean reads + SEM = 17,744,213 + 889,174, total *G. morsitans* mean reads + SEM = 17,036,187 + 850,383, Unpaired Student’s *t*-test, *p* = 0.57, Additional file 1: Figure S3A) or mapped reads (mapped *G. brevipalpis* mean reads + SEM = 10,686,667 + 823,288, mapped *G. morsitans* mean reads + SEM = 9,164,444 + 1,165,016, Mann-Whitney test, *p* = 0.42, Additional file 1: Figure S3B) between tsetse species libraries.

### *Wigglesworthia*-based analyses

As *Wigglesworthia* shapes multiple aspects of tsetse physiology, immune system maturation and metabolism [17, 26–28, 31–36], we were particularly interested in identifying genes expressed by *Wigglesworthia* within teneral flies and determining whether these may differ between sexes and tsetse species. Our rationale for these analyses is that distinct expression profiles may likely be a product of host-symbiont interdependency, representing points of diversification in the symbiosis during the course of coevolution of tsetse and their respective *Wigglesworthia* which may then impact tsetse biology including vector competence.

**Fig. 1:**
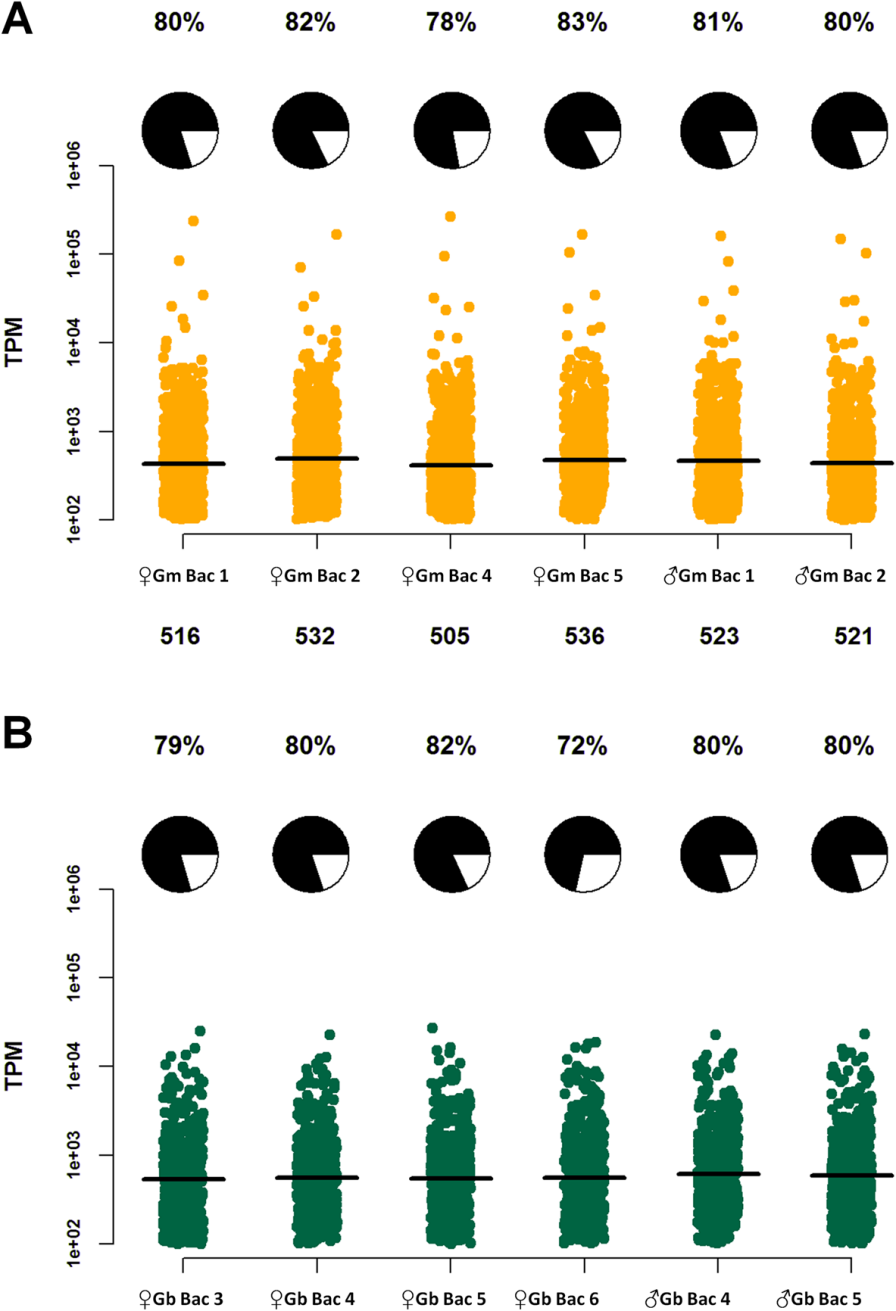
*Wigglesworthia* gene expression per tsetse species. Median TPM values (for genes with TPM > than 100) are shown as horizontal black lines for **A** *Wigglesworthia* within teneral *G. morsitans* bacteriomes and **B** *Wigglesworthia* within teneral *G. brevipalpis* bacteriomes. Dots around the median indicate TPM values of individual genes. The percentage of the total *Wigglesworthia* gene count represented by loci with TPM > 100 is indicated as pie charts on top of each corresponding library. On the x axis, the identification of the library origin and the number of *Wigglesworthia* genes with TPM > 100 is indicated. The y axis is in logarithmic scale

Our initial comparison focused on highly expressed *Wigglesworthia* genes, which we defined as loci with expression levels of > 100 Transcripts per million (TPM) (following [37–40]). Using these criteria 72–83% of the *Wigglesworthia* genome was highly expressed across all libraries indicating an active transcriptional profile for this symbiont upon tsetse adult metamorphosis (Fig. 1). Within species comparison demonstrated no significant difference in the mean *Wigglesworthia* expression between sexes for *G. morsitans* isolates (Wgm; female mean TPM + SEM = 1639 + 225.6, male mean TPM + SEM = 1562 + 252.1; Mann-Whitney test, *p* = 0.7865; Additional file 1: Fig. S4A). However, there was a slightly higher mean expression for *Wigglesworthia* genes within male bacteriomes of *G. brevipalpis* isolates (Wgb; mean female TPM + SEM = 1133 + 46.01, mean male TPM + SEM = 1239 + 67.65; Mann-Whitney test, *p* = 0.022; Additional file 1: Fig. S4B). Upon comparison of bacteriome libraries between the tsetse species, mean *Wigglesworthia* expression is significantly higher within *G. morsitans* relative to *G. brevipalpis* libraries (Mean Wgm TPM + SEM = 1613 + 172.1, mean Wgb TPM + SEM = 1169 + 38.09); Mann-Whitney test, *p* < 0.0001; Additional file 1: Fig. S4C), supporting greater *Wigglesworthia* activity within *G. morsitans* and, although speculative, higher symbiont reliance.

**Fig. 2:**
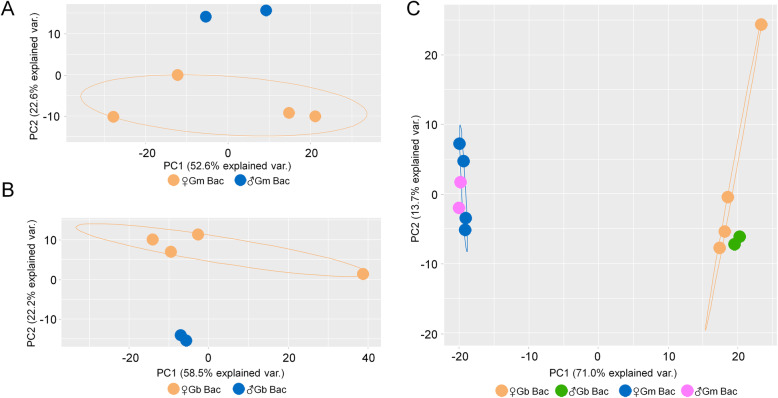
Principal component analysis (PCA) of *Wigglesworthia* gene expression based on TPM data. **A** PCA of *W. g. morsitans* genes sorted by sex, **B** PCA of *W. g. brevipalpis* genes sorted by sex and **C** PCA of 591 orthologs shared between *W. morsitans* and *W. brevipalpis genome*, sorted by sex and species. A normal data ellipse with a probability of 0.68 is shown for **A**-**C**

Principal Component Analysis (PCA) was used to compare the global gene expression of *Wigglesworthia* between female and male libraries within a host species (Fig. 2A & B). In both tsetse species, the female and male *Wigglesworthia* libraries separated along the second principal component, which explained ~ 22% of variance in expression. An additional PCA on a core gene set (consisting of 591 genes) was performed to compare *Wigglesworthia* gene expression between the tsetse species. Upon the comparison of *Wigglesworthia* expression between *G. brevipalpis* and *G. morsitans* bacteriomes, species-specific libraries separated along the first principal component, which explained 71% of variance (Fig. 2C).

**Fig. 3:**
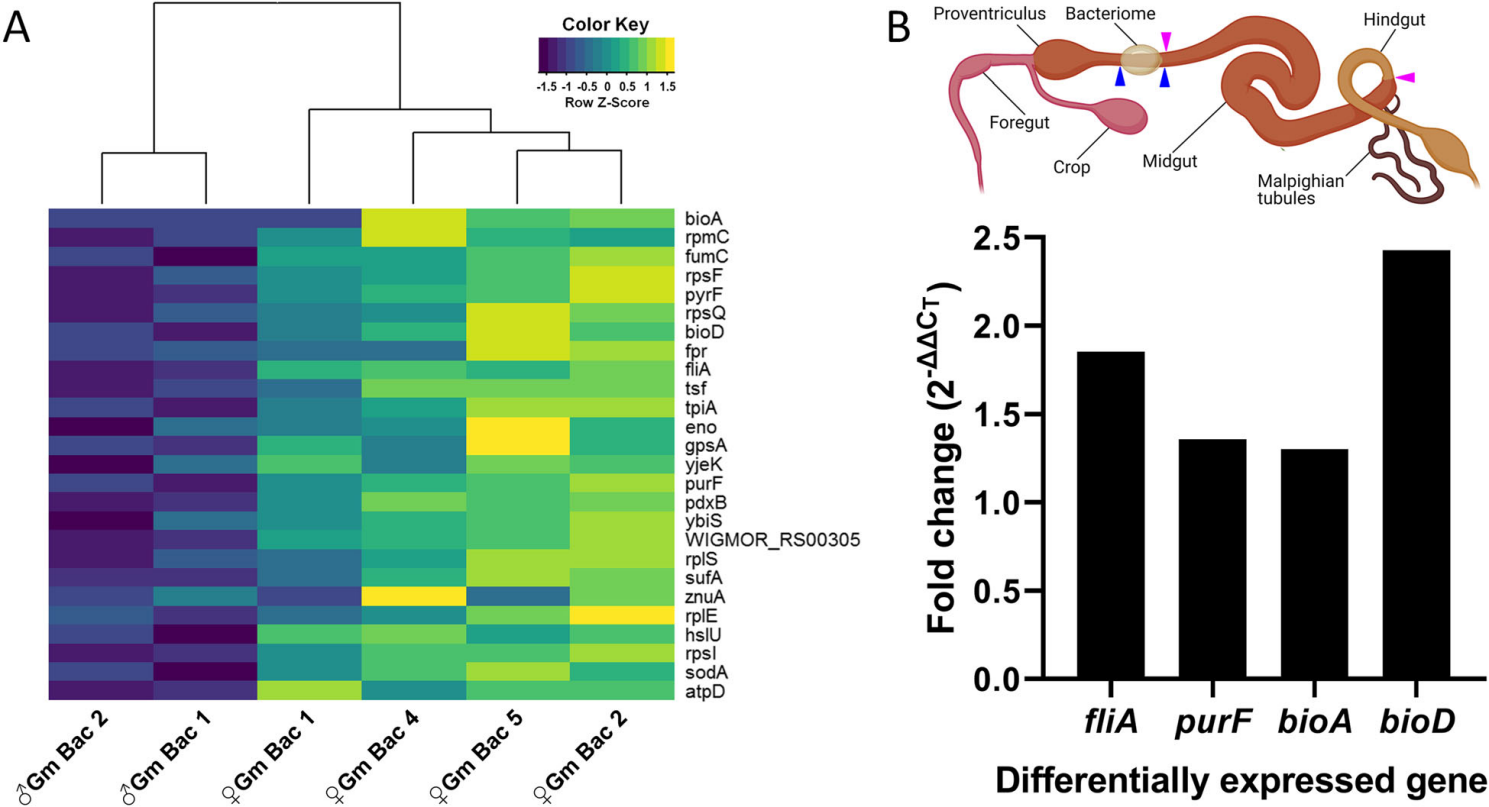
Differentially expressed *Wigglesworthia* genes between teneral males and females within *G. morsitans* bacteriome libraries. **A** A heatmap with row-normalized expression levels are shown where each row represents a gene and each cell represents the relative expression level for a sample in terms of Z-scores [observed transcripts per million (TPM) minus row mean TPM, divided by the standard deviation of TPMs for that row]. Values higher than the row mean are represented in yellow, and values lower than the row mean are represented in blue. Gene names are shown on the right. **B** Validation of selected *Wigglesworthia* genes found to be differentially expressed between *G. morsitans* isolates of females and males. Fold change as estimated by the 2^-ΔΔCt^ method via qRT-PCR supports that these genes are upregulated in females. The bacteriome is within the blue arrowheads and the midgut section is within the pink arrowheads (Created from Biorender.com)

*Tsetse sex drives differential expression of Wigglesworthia genes.* DESeq [41] was used to identify differentially expressed genes (DEGs). A total of 26 DEGs were identified between *Wigglesworthia* libraries obtained from female versus male *G. morsitans* bacteriomes (Fig. 3A, Additional file 2: Table S1), with all of these significantly upregulated within female bacteriomes. Eleven of these genes (42%) are involved in metabolic roles including B vitamin synthesis such as *bioA* and *bioD,* both components of the biotin (B1) synthesis pathway, and *pdxB* in pyridoxal 5′-phosphate (B6) metabolism. The sigma 28 regulator of class III flagella genes (*fliA*) involved in controlling the assembly of the final components of flagellum and WIGMOR_RS00305, a homolog of *fliJ,* were also significantly increased in expression. The *fliJ* gene within *Wigglesworthia* isolated from *G. morsitans* was previously characterized as a pseudogene due to truncation [42], but a 41% amino acid identity between the two *Wigglesworthia* homologs with a particularly high retention in residue identity within the two critical binding domains (aa 39–51 for FlgN binding and aa 65–82 for FliT binding) [43] suggests at least some preservation of function which merits further investigation. The gene *purF*, also significantly upregulated, encodes amidophosphoribosyltransferase, the enzyme that catalyzes the initial step in de novo purine biosynthesis [44, 45].

An elevated expression of *Wigglesworthia hslU*, a homolog of a chaperone-related protease [46, 47], was also observed within *G. morsitans* female bacteriomes. Interestingly, the oligomerization of HslU subunits is associated with the regulation of cell growth [48] and may support the significant increase in *Wigglesworthia* density during early adulthood observed in females but lacking in male tsetse flies [30]. We further confirmed the upregulation of a subset of these *Wigglesworthia* genes (*fliA, purF, bioA* and *bioD*) within female bacteriomes through qRT-PCR (Fig. 3B). Although PCA analyses showed a sex separation between *G. brevipalpis* libraries, DESeq found no significant difference on a gene by gene basis when comparing *Wigglesworthia* expression.

**Fig. 4:**
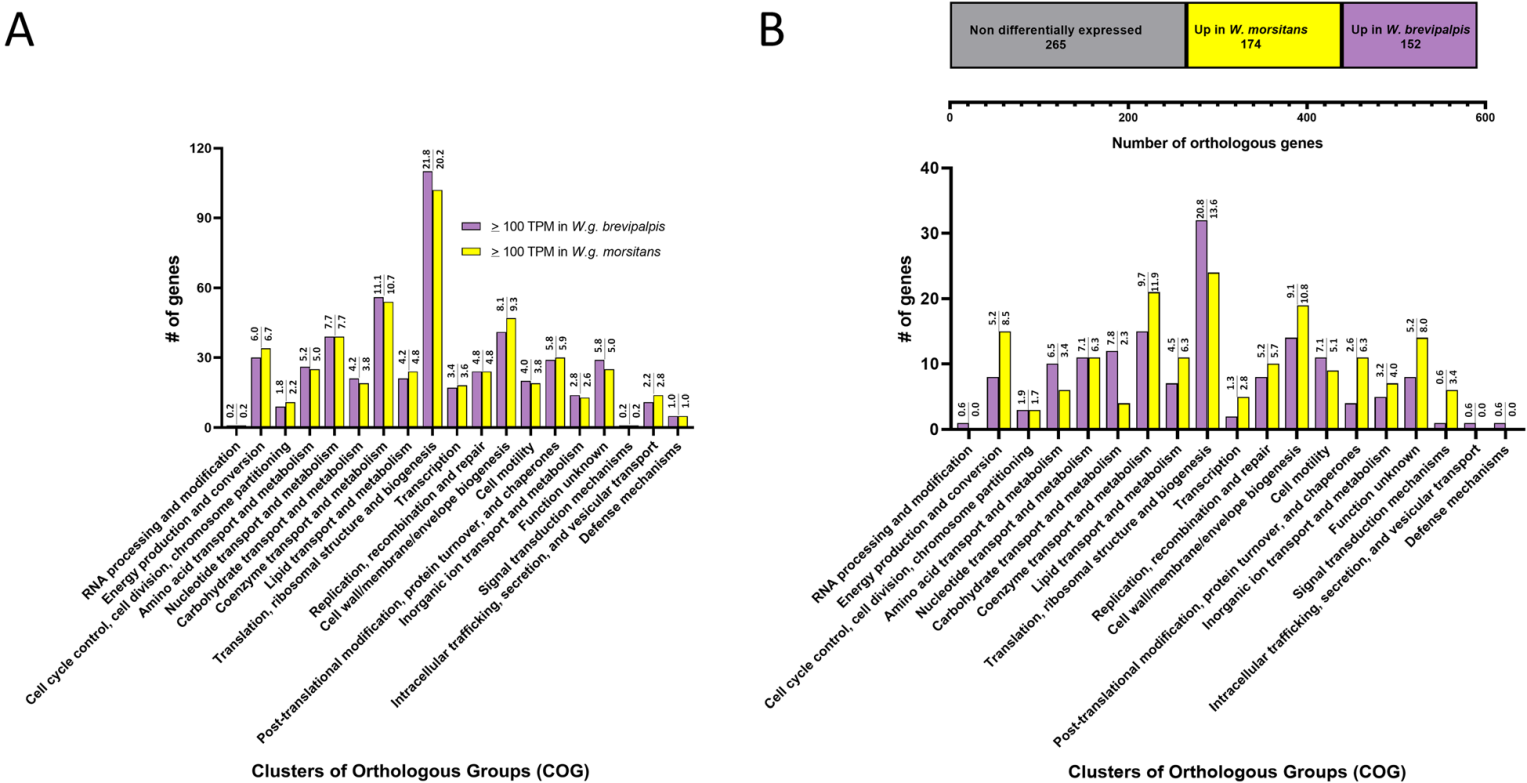
COG classification of highly expressed genes and DEGs between *Wigglesworthia* isolates. **A** Clustering of highly expressed genes (TPM > 100) into orthologous groups. Each COG shows columns for highly expressed genes in each *Wigglesworthia* isolate. The numbers on top of the bars indicate the percentage of genes included in that particular COG relative to the total number of genes with TPM > 100 for each *Wigglesworthia* isolate. **B** Top, the horizontal bar partitions the fractions of non-differentially and differentially expressed genes among the 591 orthologs shared between the two *Wigglesworthia* isolates. Bottom, clustering of the differentially expressed genes into orthologous groups. Each COG shows columns for genes upregulated in each *Wigglesworthia* isolate. The numbers on top of the bars for each *Wigglesworthia* isolate indicate the percentage of genes included in that particular COG relative to the total of differentially expressed genes (*n* = 326). If a gene had more than one COG, it was placed into each respective COG (i.e., these genes have more than one representation). There are three categories in which the *Wigglesworthia* isolate from *G. morsitans* did not have genes that were significantly upregulated

*Differential expression of* Wigglesworthia *genes between tsetse species.* Within the 16 Clusters of Orthologous Groups (COG, [49]) that were shared between the two tsetse species, the proportion of genes within each COG did not significantly differ, neither for the highly expressed genes (TPM > 100, Kolmogorov-Smirnov test, *p* > 0.9999, Fig. 4A), nor for the DEGs (Kolmogorov-Smirnov test, *p* = 0.9718; Fig. 4B). An active expression profile was observed for *Wigglesworthia* immediately following host adult metamorphosis. Specifically, ‘translation, ribosomal structure and biogenesis’ followed by ‘coenzyme transport and metabolism’ were the most enriched COGs, highlighting the preservation of *Wigglesworthia*’s vitamin provisioning role following host speciation. A total of 326 orthologs (55% of 591 protein coding genes, Fig. 4B) were identified as DEGs between the *Wigglesworthia* isolates of the two tsetse species (Additional file 3: Table S2). The *Wigglesworthia* symbionts within the *G. morsitans* bacteriomes demonstrate upregulation of 174 genes (53% of DEG genes, which corresponds to 29% of orthologous genes), while 152 genes (47% of DEG genes, which corresponds to 26% of orthologous genes) are upregulated within *G. brevipalpis* (Fig. 4B). Three COG categories which were unique to *G. brevipalpis* each only housed a single DEG that was significantly upregulated; “RNA processing and modification” (*yjeR*), “Intracellular trafficking, secretion and vesicular transport” (*fliI*), and “Defense mechanisms” (*yadH*). Both *Wigglesworthia* isolates (Fig. 4B) had a significant proportion of differentially expressed genes (i.e. 22.1% of DEG) falling within COGs associated with “Energy production and conversion” and towards the “Transport and metabolism of …” “lipids”, “amino acids”, and “carbohydrates”. Additionally, ~ 11% of DEGs fell in the COG of “Coenzyme transport and metabolism”.

**Fig. 5:**
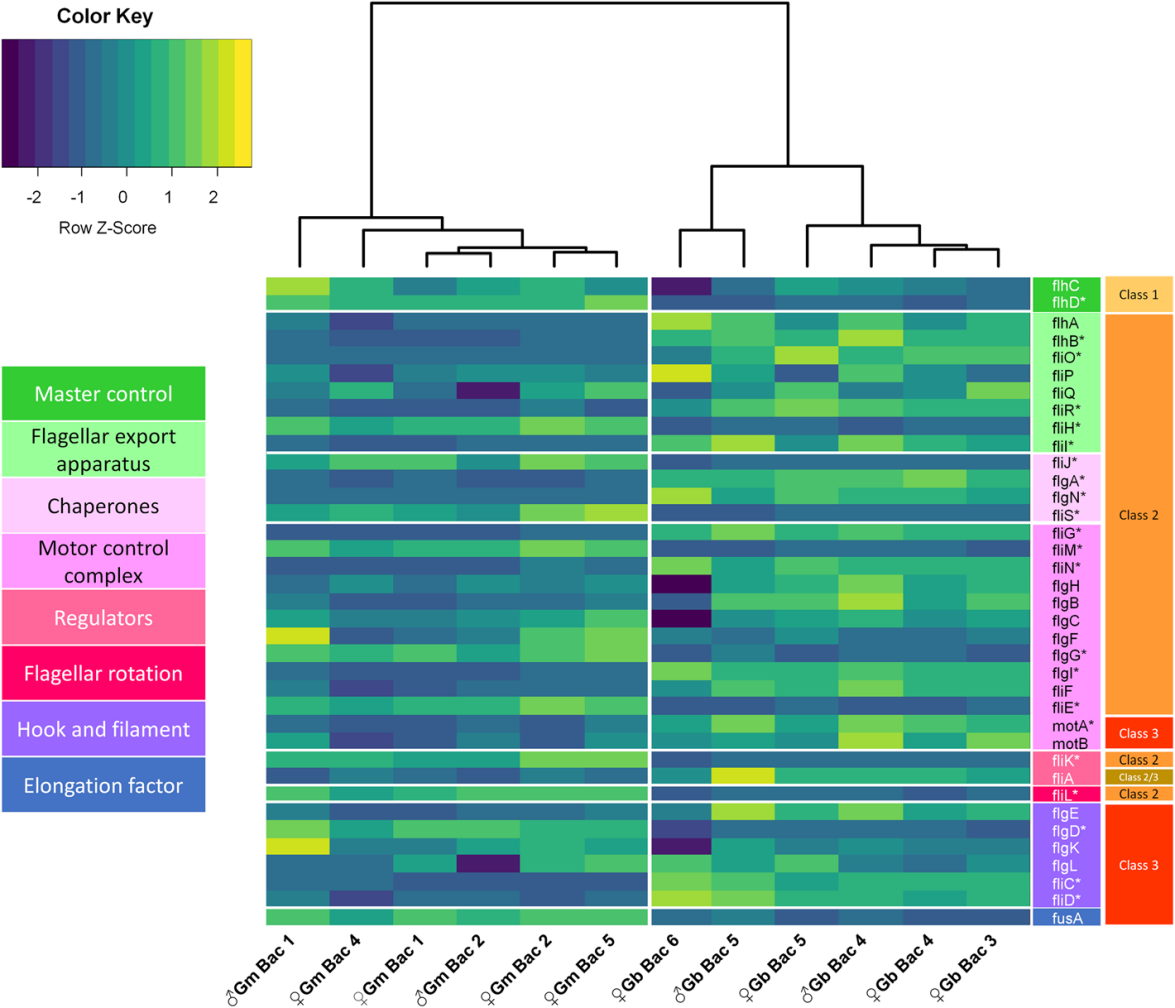
Distinct expression patterns of *Wigglesworthia* flagellar genes are characteristic within a tsetse host species. A heatmap comparing the expression of flagellar genes in the *Wigglesworthia* isolates from *G. brevipalpis* and *G. morsitans* bacteriomes. Genes are vertically organized by function and class which are indicated by the colored blocks at the right. Asterisks adjacent to the gene names indicate statistically significant differences in expression between the *Wigglesworthia* isolates of the two species

The *Wigglesworthia* genomes encode a complete flagellar apparatus [42, 50] which likely facilitate its evolutionary persistence through vertical transmission using a milk gland route [7–9]. Interestingly, the expression patterns of flagellar genes cluster by host species. A total of 22 genes out of 37 flagellum genes examined (~ 60%) significantly differed in their expression between the tsetse species, with one belonging to Class I, 18 to Class II and three to Class III (Fig. 5). The operon *flhDC* is a master regulator of flagellar genes [51] that activates the expression of Class II flagellar components. A significantly higher expression of the *Wigglesworthia flhDC* operon was observed in *G. morsitans* isolates*.* Counterintuitively, the corresponding expression levels of Class II flagellar genes downstream to *flhDC* were downregulated. Previous tsetse-*Wigglesworthia* studies demonstrated that the flagellar apparatus is downregulated within the bacteriome population, while it is actively synthesized by the milk gland population [42]. Interestingly, that previous study was based on the detection of the genes *fliC* and *motA,* which have a lower expression in our *G. morsitans* bacteriomes isolates, but a significantly higher expression in our *G. brevipalpis* isolates. A differential spatial and temporal regulation of flagella components of *Wigglesworthia* between tsetse host species merits further investigation.

### *Sodalis-*based analyses

**Fig. 6:**
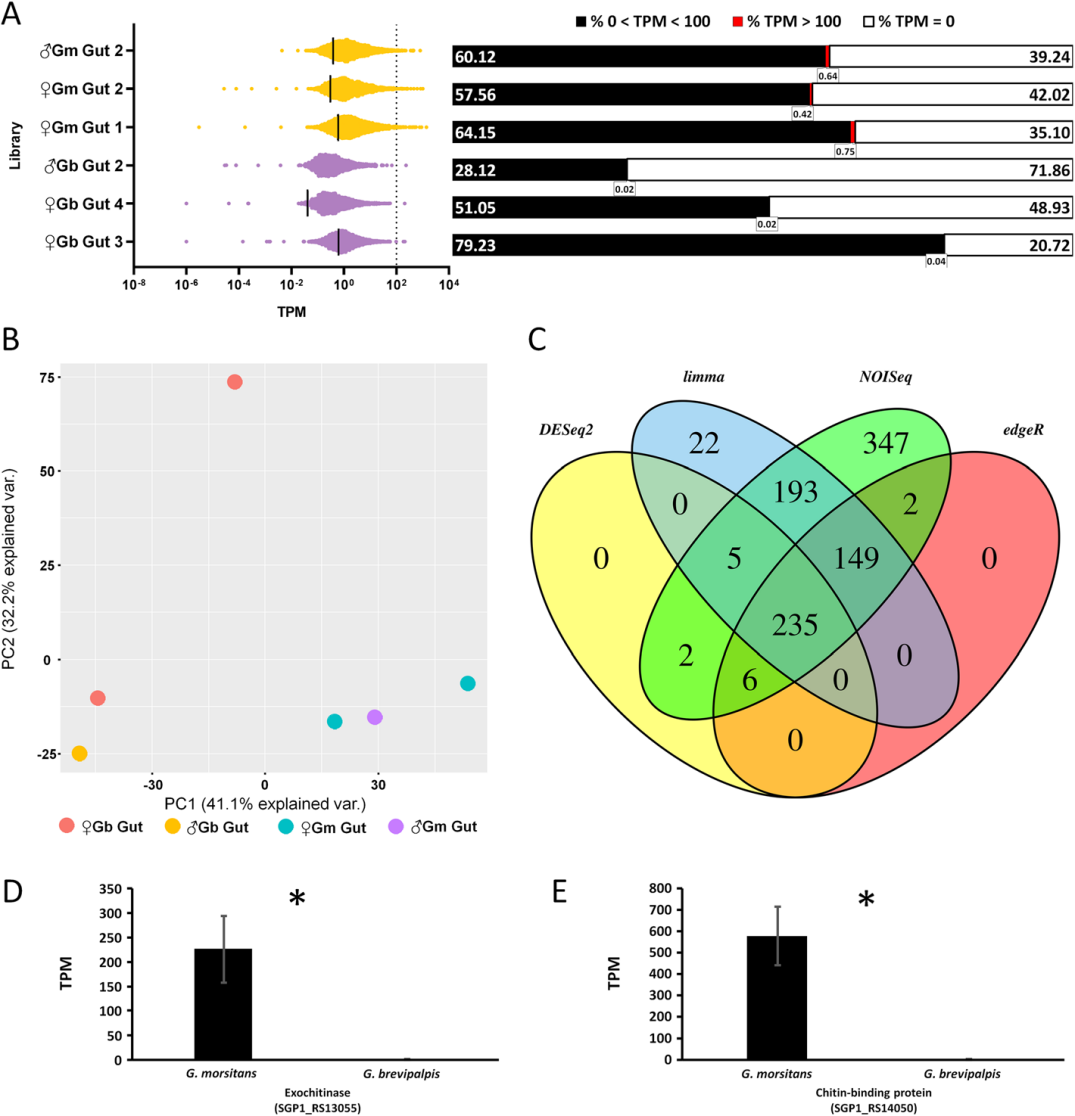
*Sodalis* transcriptomic profiles within teneral tsetse flies. **A** Scatterplot with TPM distribution within each tsetse midgut library; x axis is in log_10_ scale, only genes with TPM > 0 are plotted (left). Dots around the median indicate TPM values of individual genes. Bar graphs partition the percentage of the total number of genes according to their expression levels (low expression < 100 TPM, high expression > 100 TPM or no expression with TPM = 0) with corresponding percentage of genes with > 100 TPM indicated by the call-outs on the bottom of each corresponding bar (right). Across midgut libraries of both tsetse species, the *Sodalis* isolates exhibit low gene expression. **B** PCA analysis indicates that 41.1% of the variability across *Sodalis* expression may be accounted for by tsetse host species. **C** Output of differential expression analyses to obtain the consensus set of DE genes between tsetse species isolates. The intersect at the center of the Venn diagram contains the genes found to be differentially expressed by the four informatic approaches indicated on each oval (*n* = 235). **D**-**E** Average TPM from three gut RNASeq libraries of selected *Sodalis* genes involved in the metabolism of chitin; asterisks indicate significant differential expression (*; *p* < 0.0001)

To further test the hypothesis that host species impact transcriptomic profiles in their microbiota and that these will be more pronounced the older the symbiosis, we also characterized and compared *Sodalis* gene expression in the two tsetse species. In all our midgut libraries, the reads mapping to the *Sodalis* genome constitute a very small proportion of total reads (~ 0.1%). The percentage of genes that show some level of expression varies widely across libraries, ranging from 58 to 64% in the *G. morsitans* midgut isolates, with an even greater span in the *G. brevipalpis* midgut isolates of 28–79% (over a total of 4541 protein coding genes) (Fig. 6A). If a gene was expressed, it likely had a transcription level of < 100 TPM (i.e. > 99% of 4541 genes, Fig. 6A). Only a small number of genes, constituting less than 1% of the total genes, is highly expressed (TPM > 100). More specifically, the proportion of genes that were highly expressed is significantly larger in the *G. morsitans* isolates (26.33 + 4.410) when compared to *G. brevipalpis* isolates (1.333 + 0.333, Welch’s test, *p =* 0.0291, Additional file 4: Table S3). For those genes with > 100 TPM, the average TPM does not differ between the two tsetse host species (275.9 + 26.87 in *G. morsitans* isolates vs. 208.2 + 9.799 in *G. brevipalpis* isolates, nested *t-*test, *p* = 0.6042). Further, a PCA analysis shows that tsetse host species can account for 41% of gene expression variation (PC1) between the *Sodalis* isolates (Fig. 6B), with no clustering by host sex. The analysis of read counts via IDEAmex [52] identified 235 genes to be differentially expressed between *Sodalis* isolates of the two tsetse species (Fig. 6C, Additional file 5: Table S4). These genes represent a very small proportion of the total protein coding genes (5.2%), particularly when contrasted with the high fraction of orthologs differentially expressed between the two *Wigglesworthia* isolates (55%).

#### Sodalis exochitinase expression profile may contribute to tsetse vector competence

The higher susceptibility of teneral tsetse to trypanosome infections is thought to partly arise due to the immaturity of the peritrophic matrix (PM). The PM of adult tsetse is continuously produced by the cardia (characteristic of a Type II PM) and forms a protective semipermeable barrier within the intestinal tract by surrounding the blood bolus within an endoperitrophic space. Due to the PM being rich in chitin [53] coupled with the higher susceptibility of tsetse flies towards trypanosome infection as tenerals [54], *Sodalis* genes encoding chitin-associated proteins may compromise PM integrity and thereby facilitate trypanosome infection [55–57]. Two genes involved in chitin-associated activities were among the most differentially expressed between the two *Sodalis* isolates (Fig. 6D and E). The genes encoding the predicted chitin-binding protein (NCBI Protein ID: BAE74790) and exochitinase (*chiA;* NCBI Protein ID: BAE74749) are significantly upregulated within the *Sodalis* isolate of *G. morsitans* relative to that of *G. brevipalpis* during the teneral host stage (Fig. 6D and E). The exochitinase gene is essential for *Sodalis* persistence within tsetse [58] as its chitinolytic activity produces *N-*acetyl-D-glucosamine (Glc*N*Ac) which is the principal carbon source for this bacterium. Additionally, the Glc*N*Ac monosaccharides inhibit anti-trypanosomal lectins present in tsetse midgut [59] impeding their binding to trypanosome surface carbohydrates promoting parasite establishment [60]. The *Sodalis* exochitinase activity may also facilitate trypanosomes crossing into the ectoperitrophic space by disrupting the physical integrity of the PM.

*Highly expressed* Sodalis *genes contain a large proportion of DEGs.*

**Fig. 7:**
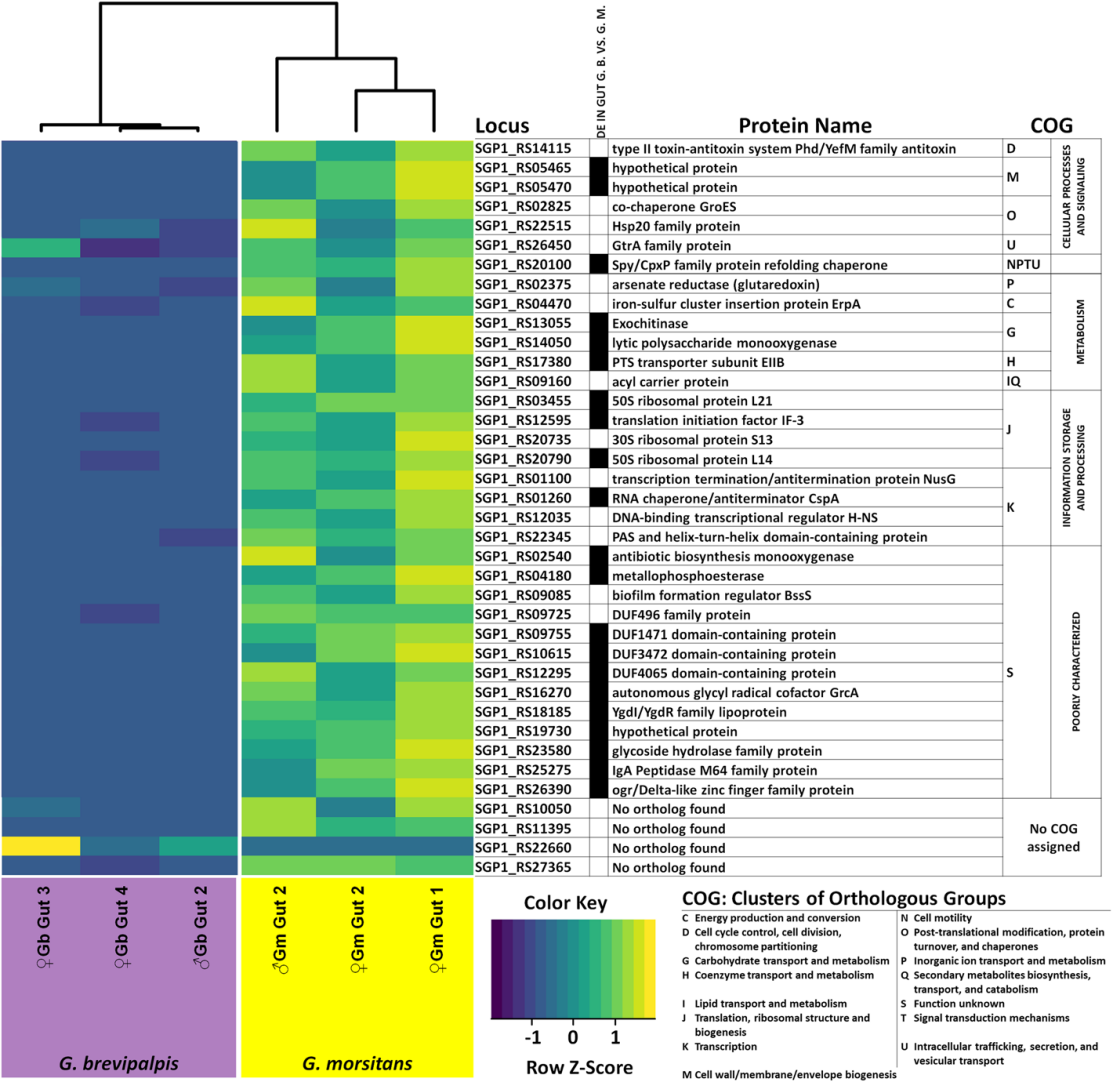
Highly expressed genes in the *Sodalis* transcriptome of two tsetse species. Heatmap of *Sodalis* genes that have a high expression (TPM > 100) in at least one library. The columns at the right show the locus identifier, a black square for significant differential expression between midgut libraries of *G. brevipalpis* and *G. morsitans* isolates, a brief description of the coded gene, and the COG classification. Genes for which eggNOG mapper found no orthologs, are found at the bottom of the heatmap

Interestingly, the highly expressed genes (TPM > 100) in the *Sodalis* transcriptome contain also a disproportionately high number of DEGs (Fig. 7). As mentioned before, only about 5.2% of all *Sodalis* coding sequences are differentially expressed between the host species isolates; however, the subset of genes with TPM > 100 (Fig. 7) contains 55.3% of the total DEGs, which may indicate genes important for the *Sodalis-*tsetse symbiosis. For example, orthologs of type II toxin-antitoxin systems (SGP1_RS14115) along with a formation regulator BssS (SGP1_RS09085) facilitate biofilm formation [61, 62]; hypothetically this may support close proximity of *Sodalis* to the PM lining of the gut lumen, where the bacterium may then deploy exochitinase (SGP1_RS13055), likely aided by lytic polysaccharide monooxygenase (SGP1_RS14050) and glycoside hydrolase family protein (SGP1_RS23580), in order to degrade chitin [63, 64] for access to Glc*N*Ac as nutritional source. Although the main known role of chaperones is towards correcting misfolded proteins during stress response [65–68], they are also hypothesized to be important in mediating insect-bacteria interactions [69]. For example, *groES* constitutes one of the most abundant transcripts of the ant symbiont *Blochmannia* [70], *Wigglesworthia* in wild tsetse populations [71]) and *Buchnera* within aphids [72].

### Tsetse fly based analyses

Multiple studies have demonstrated that trypanosome and bacterial infections in tsetse flies involve the expression of antimicrobial peptides (AMPs), reactive oxygen species (ROS) and other key anti-pathogen genes [73–77]. Yet, little has been done to contrast immunological profiles at specific developmental timepoints between tsetse species and integrating with the activity of the core microbiota. To complement the characterization of the symbiont transcriptomes, and to obtain a holistic picture of the genomic interplay within tsetse that may enhance our understanding of interspecific differences in host traits, we also examined the transcriptomes of *G. brevipalpis* and *G. morsitans*.

#### Tsetse transcriptomic profiles show a distinct clustering by species and tissues

**Fig. 8:**
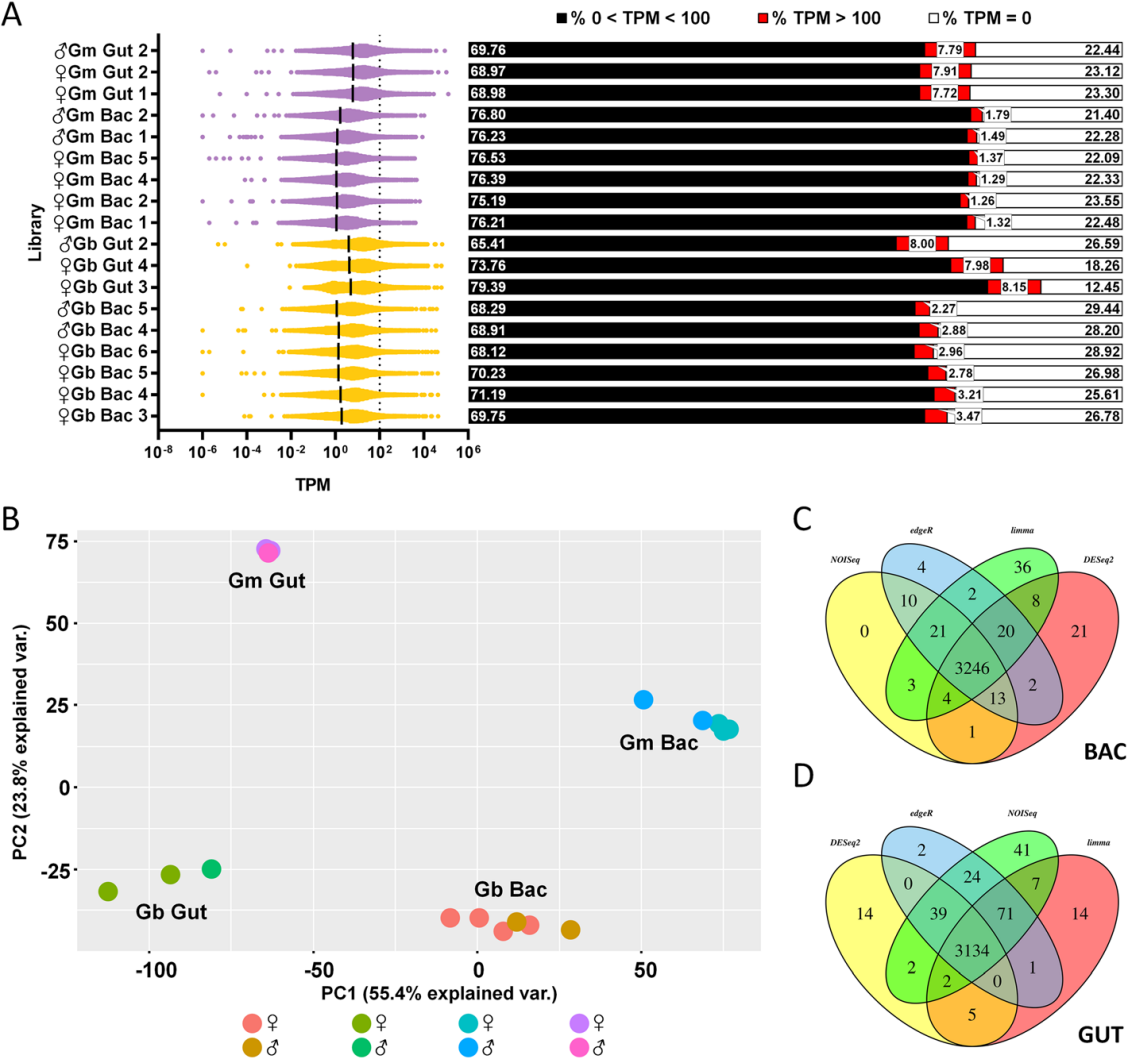
Transcriptomic profiles of tsetse genes within teneral tsetse flies. **A** Scatterplot with TPM distribution within each tsetse library; x axis is in log_10_ scale, only genes with TPM > 0 are plotted (left). The median is indicated with a vertical black line, TPM of 100 is indicated with dotted line; bar graphs partition the percentage of the total number of genes according to their expression levels (low expression < 100 TPM, high expression > 100 TPM or no expression with TPM = 0). The call-outs on the side of each corresponding bar (right) indicate the percentage of genes with > 100 TPM. Black dots at the left and right indicate the median and the cutoff of 100 TPM, respectively. **B** PCA analysis indicates that 55.4% of the variability across tsetse expression may be accounted for by tsetse tissue (PC1), while 23.8% of the variability may be accounted for by tsetse species (PC2). **C** Output of IDEAmex which depicts DE genes within the bacteriomes of tsetse species isolates; the intersect at the center of the Venn diagram contains the tsetse genes found to be differentially expressed by the four informatic approaches as identified adjacent to each oval; *n* = 3246 DE genes. **D** Output of IDEAmex which depicts DE genes within the midguts of tsetse species isolates; the intersect at the center of the Venn diagram contains the tsetse genes found to be differentially expressed by the four informatic approaches as identified adjacent to each oval; *n* = 3134 DE genes

If a tsetse gene was expressed, it likely had a transcription level of < 100 TPM (Fig. 8A). Only a relatively small proportion (~ 8.2%) of the total gene count is highly expressed (TPM > 100); however, this number is significantly larger in the *G. brevipalpis* isolates [bacteriomes = 435.7 + 24.65 (*n* = 6 libraries); midguts = 1197 + 8.212 (*n* = 3 libraries)] when compared to *G. morsitans* [bacteriomes = 183.2 + 10.45 (n = 6 libraries); midguts = 1007 + 7.265 (n = 3 libraries)] in both bacteriomes and midgut libraries (*t*-test, *p* < 0.0001). When looking at specific groups of libraries, for genes with > 100 TPM within midguts, the average TPM does not differ between the two tsetse host species [685.0 + 44.69 (*n* = 3590 genes) in *G. brevipalpis* vs. 737.6 + 70.59 (*n* = 3020 genes) in *G. morsitans* isolates, nested *t-*test, *p* = 0.5158]. However, when comparing genes with > 100 TPM within bacteriome libraries, the average TPM is significantly higher for *G. brevipalpis* (overall average TPM + SEM of 675.9 + 52.98, *n* = 2614 genes) in comparison to *G. morsitans* (465.1 + 25.45, *n* = 1099 genes, nested *t-*test, *p* = 0.0115). PCA analysis shows that 55.4% of the variability across tsetse gene expression is explained by tsetse tissue (PC1), while 23.8% of the variability may be accounted for by tsetse species (PC2, Fig. 8B), with no clustering by fly sex. Differential expression analyses via IDEAmex shows 3246 DEG between *G. brevipalpis* and *G. morsitans* in bacteriome libraries (Fig. 8C, Additional file 6: Table S5) and 3134 DEG between *G. brevipalpis* and *G. morsitans* in midgut libraries (Fig. 8D, Additional file 7: Tables S6).

#### Tsetse species differ in the expression of immunity related pathways as tenerals

**Fig. 9:**
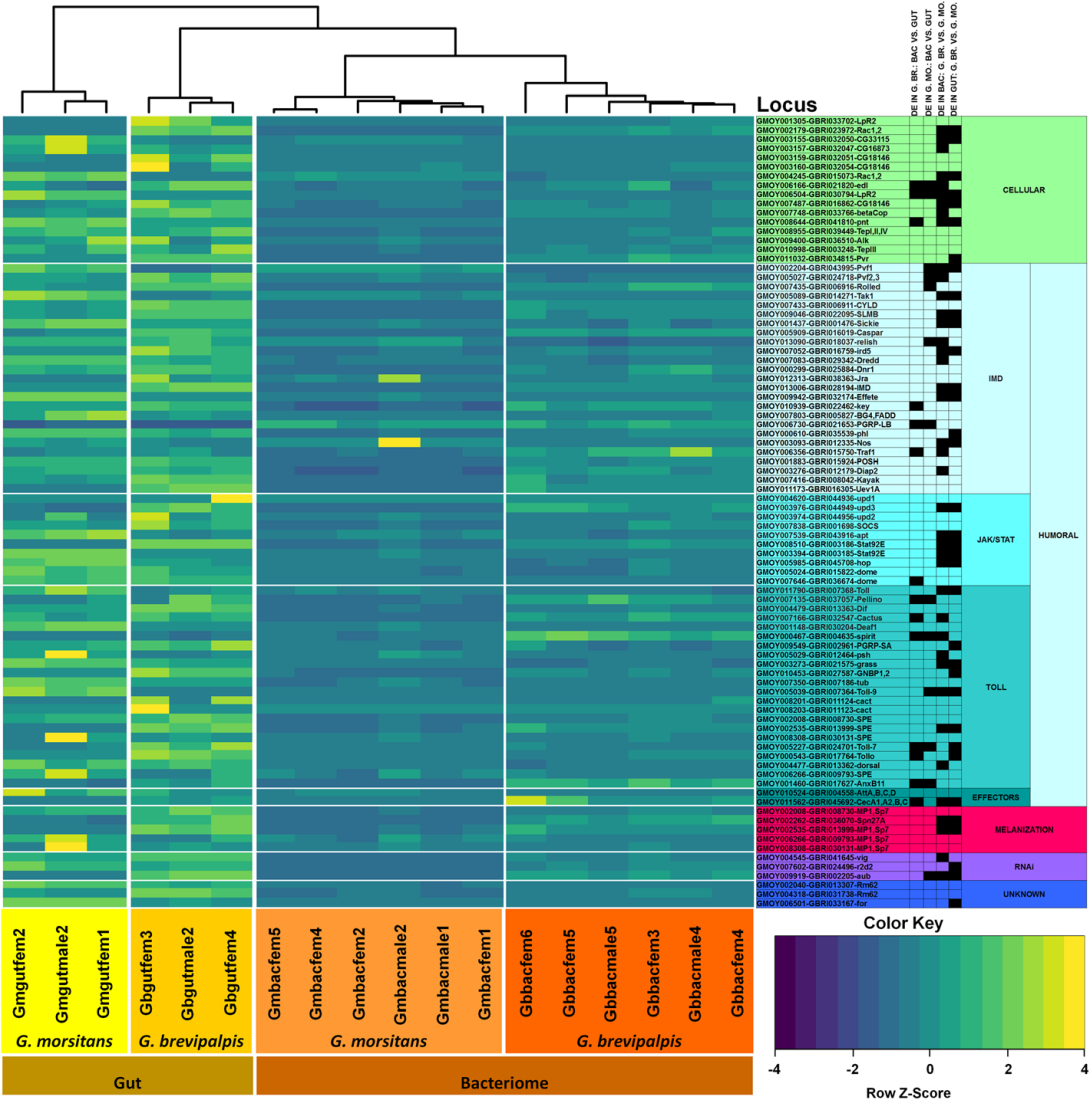
Expression profile of immunity genes in teneral *G. morsitans* and *G. brevipalpis*. Heatmap with row-normalized expression levels are shown where each row represents a gene and each cell represents the relative expression level for a sample of midguts or bacteriomes in terms of Z-scores [observed transcripts per million (TPM) minus row mean TPM, divided by the standard deviation of TPMs for that row]. Values higher than the row mean are represented in green, and values lower than the row mean are represented in red. VectorBase gene ID for the two tsetse species is provided at the right of each row, including the gene symbol for the ortholog in *D. melanogaster* according to FlyBase. A black square next to the gene indicates a significant differential expression (adjusted *p* < 0.05) according to the comparison on the column headers located at the: DE = differentially expressed; G. BR. = *G. brevipalpis;* G. MO. = *G. morsitans;* BAC = bacteriome; GUT = midgut. The *p*-values for the corresponding comparisons are included in Supplementary Additional file 8: Table S7 and Additional file 9: Table S8. Blocks at the right group genes by immunity class, the unknown category indicates genes that were differentially expressed in *D. melanogaster* upon challenge with pathogenic bacteria, but are not genes associated with the other classes, immunity classes are provided only as a guidance, as cross-talk between pathways exists; blocks at the bottom indicate tsetse tissue and species of origin

Given that immunity plays an essential role in mediating a spectrum of host-microbe interactions [78, 79], we compared the immunological transcriptomes of *G. brevipalpis* and *G. morsitans* bacteriomes and midguts. Immunity-related genes were identified via orthology with *D. melanogaster* [80] and interspecies comparison performed [81]. These genes are critical components of various immunological responses including cellular, humoral, melanization and RNAi pathways (as identified in [80]). A heat map of immunity gene expression demonstrates clustering that distinguishes guts from bacteriomes and also by tsetse species (Fig. 9, Additional files 8 and 9: Tables S7 and S8 respectively). On average, genes involved in the various immunological mechanisms, are upregulated in midguts relative to the bacteriomes supporting the immunopermissive space of the bacteriome serving to protect essential *Wigglesworthia* symbionts (37.61 + 3.442 TPM in midguts vs. 15.88 + 1.154 TPM in bacteriomes; *n* = 86, nested t-test, *p* < 0.0001). This immune tolerance is further exemplified by the expression of *pgrp-lb* which is significantly higher within the bacteriomes of both tsetse species relative to midguts. Within the bacteriome the peptidoglycan recognition protein-LB (PGRP-LB) scavenges peptidoglycan released during *Wiggleworthia* cell division, thus preventing the activation of the hostile IMD pathway and offering symbiont protection [36, 82].

The cellular immunity category contains the only DEG identified between intraspecific bacteriomes and midguts and also between tsetse species, *LpR2* (GMOY006504, GBRI030794). *LpR2* has a significantly higher expression in the midgut libraries of both tsetse species when compared to the corresponding bacteriome expression levels. Interspecies comparisons also indicate that *LpR2* is significantly upregulated within *G. morsitans* midguts and bacteriomes, when compared to the corresponding *G. brevipalpis* libraries. *LpR2* encodes a lipophorin receptor involved in the regulation of the Toll pathway [83]. An additional cellular immunity gene expressed significantly higher in *G. brevipalpis* midgut libraries was *pvr* which coordinates immunity responses through the inhibition of humoral immunity while stimulating hemocyte distribution, an early event in cellular immunity [84].

Humoral immunity pathways, such as Toll and Imd, are generally activated upon infection by Gram-positive and Gram-negative bacteria, respectively, and lead to the production of distinct sets of AMPs, such as drosomycin, defensin and metchnikowin (Toll pathway) and attacin, cecropin and diptericin (Imd pathway) [85–89]. The *imd* gene is highly expressed both in *G. brevipalpis* midguts and bacteriomes. The gene *imd* is a strong regulator of antimicrobial responses against invading Gram-negative bacteria by inducing the expression of transcripts that encode antimicrobial effector proteins upon the recognition of specific microbial-associated molecular patterns [90], which is also consistent with the higher expression of *cecropin* within *G. brevipalpis* midguts [91]. A higher expression of *cecropin* may confer greater protection to *G. brevipalpis* against trypanosome infections, as products of orthologs in other insect species have killing activity against the related *Trypanosoma cruzi* [92, 93].

Interestingly, *effete* is highly expressed within midguts, but it is particularly higher in *G. morsitans.* The *effete* protein (*Ubc5*) mediates the polyubiquitination of IMD*,* leading to its degradation within the proteasome [94] which aligns with a lower *imd* expression. UevA joins *Ubc5* in the polyubiquitination of IMD [94]; *Uev1A* is highly expressed across libraries, however its expression is not significantly different between any of the library comparisons. These results may suggest a decreased potency of the IMD pathway in *G. morsitans* in the teneral state, which merits further investigation*.*

Within the Toll pathway, *G. brevipalpis* bacteriomes have a higher abundance of *spirit* transcripts in comparison to *G. morsitans.* The serine protease *Spirit* functions as a processing enzyme for the cytokine-like molecule Spätzle [95], a required initial step towards the activation of the Toll receptor for countering Gram-positive and fungal pathogens. The Toll pathway inhibitors *pellino* and *cactus* are upregulated in *G. brevipalpis* bacteriomes when compared to midguts. The proteins *Pellino* [96] and *Cactus* [97]) inhibit the Toll pathway by impeding signal transduction from the cell surface [96]) and by decreasing transcription of antimicrobial peptide coding genes [98], respectively. This may suggest that *G. brevipalpis* flies offer a more permissive microenvironment for bacterial growth in their bacteriomes. Additionally, *annexin B11* (*AnxB11*) was also upregulated in the bacteriomes of both species. Mammalian annexins are inhibitors of immune responses, where they suppress inflammatory responses during apoptosis [99, 100]. This observation further supports a more tolerant microenvironment in both tsetse species *G. brevipalpis* and *G. morsitans* likely aimed to sustain high bacterial densities within bacteriomes when compared to midguts.

Overall, genes involved in the JAK/STAT pathway show a low expression (TPM < 100) at the teneral stage [12.62 + 1.401 TPM in JAK/STAT genes (*n* = 10) vs. 23.12 + 1.405 TPM in all immune-related genes (*n* = 86); nested *t*-test, *p* = 0.0403], however, comparative functional studies will be needed to validate differences in activation of the pathway between tsetse species. The cytokine *Upd3* mediates cellular immune response and is an activator of the JAK/STAT pathway [101]. Expression levels for *upd3* are consistently higher in *G. brevipalpis* bacteriome and midgut libraries relative to *G. morsitans*. Concurrently, the orthologs of the Signal-transducer and activator of transcription protein at 92E (*stat92E*) (GMOY008510 and GBRI003186) exhibit a higher expression in *G. brevipalpis* midguts. Upon pathogenic bacterial infection, Stat92E translocates to the nuclei of fat body cells where it drives antimicrobial peptide expression [102]. In contrast, a feedback inhibitor of the JAK/STAT pathway *Apontic* (*apt*) [103]) has relatively lower expression within *G. brevipalpis* libraries. It is puzzling that the ortholog set of the *stat92E* gene (GMOY003394 and GBRI003185) shows an opposite pattern of expression, where these have a higher level in *G. morsitans* bacteriomes and midguts when compared to the corresponding libraries within *G. brevipalpis*. This divergence in expression highlights the importance of functional characterization of genes across tsetse species.

In the melanization branch of immunity, both the Melanization Protease 1 [104] and Serine protease 7 (MP1-Sp7) may catalyze the initial activation of the pathway by cleaving the prophenoloxidase (PPO) zymogen to its active form phenoloxidase [105]. The serpin Spn27A is a serine protease inhibitor that negatively regulates melanization to limit the response to only the site of injury or infection preventing self-harm from excessive induction [106]. Interestingly, *G. brevipalpis* exhibits a simultaneously higher transcript abundance of both MP1-Sp7 (ortholog pair GMOY002535-GBRI013999) and Spn27A, which may allow this fly species to be poised to better respond should a pathogenic invasion occur and melanization required. Four tsetse ortholog pairs mapped to MP1-Sp7 in *D. melanogaster*, but the ortholog pairs GMOY002008-GBRI008730, GMOY006266-GBRI009793, and GMOY008308-GBRI030131 do not show a significant differential expression between tsetse species.

## Discussion

### Simultaneous host and symbiont examination enhances the understanding of their integrative biology

RNAseq enables the parallel assessment of transcriptomes from co-occurring species (many of which are unable to be separated without compromising the vitality of one or more partners) such as hosts and their microbiota. This approach is especially suitable for the discovery of novel points of interaction in host-microbe symbioses and the synthesis of robust hypotheses pertaining to how traits may be generated through host/microbiota activities. In this study, our main objective was to identify differentially expressed host and symbiont bacteria genes, between two tsetse fly species at the teneral stage, and to characterize how these transcriptional profiles may impact host biology including vector competence. Additionally, we hypothesized that symbionts (i.e. *Wigglesworthia*) with a lengthier host coevolution would show more distinctions in gene expression between tsetse species than recently acquired symbionts (i.e. *Sodalis*) which likely represent a greater extent of host adaptation.

The tsetse fly is recognized as a valuable animal model for enhancing our understanding of host-microbe symbiosis while also having high public health and agricultural significance as the obligate vector of African trypanosomes. As tenerals, tsetse have the highest susceptibility to trypanosome infections [25, 54, 107–110] likely due to a compilation of low levels of anti-trypanosomal binding midgut lectins, depleted fat reserves following metamorphosis, and the immature structural integrity of the PM crucial for both physical containment of trypanosomes within the endoperitrophic space and also towards midgut epithelial immune regulation [53, 60, 111]. Furthermore, vector competence varies between tsetse species with members of the *Morsitans* subgenus, including *G. morsitans,* exhibiting higher susceptibility to trypanosome infections, while those of the *Fusca* subgenus, such as *G. brevipalpis*, are comparatively poor vectors [112–114]. A deeper understanding of the molecular interactions between tsetse and its endogenous microbiota and how these may facilitate the establishment of trypanosomes during the teneral stage offer pillars for the development of novel and specific vector control strategies.

We expected to find that genes and pathways promoting trypanosome infection as tenerals would be enriched in expression in *G. morsitans* relative to *G. brevipalpis* which would associate a characteristic transcriptome profile of tsetse and its symbionts with vector competence. These interspecific distinctions may be further studied to understand functional diversification and relevancy towards tsetse biology and ecology. For example, differential transcriptomic profiles may help distinguish degrees of reliance among symbionts and tsetse species, likely influenced by differences in symbiont history with tsetse, and help to identify targets for disruption of fly-symbiont interactions in the context of novel vector control strategies targeting critical aspects of symbiosis.

The *Wigglesworthia* symbiont is not only essential for tsetse fly biology, but also its interaction with other members of the microbiota including *Sodalis* [115] and trypanosomes [27, 31]. Intriguingly, between 72 and 83% of *Wigglesworthia* genes are expressed within teneral tsetse flies indicating high activity following adult metamorphosis. The acquisition of nutrients by trypanosomes must be strategically orchestrated with host metabolism requiring a fine balance between obtaining sufficient nutrients to complete its lifecycle but not sacrificing tsetse fitness to the extent that transmission to a naïve vertebrate host is compromised. Trypanosomes are auxotrophs for metabolites that are also essential for the fly and are available in very low amounts within blood, such as B vitamins [116, 117]. For example, *G. brevipalpis* harbors a *Wigglesworthia* isolate incapable of folate (B9) biosynthesis for which trypanosomes are also deficient. The exogenous supplementation of the *G. brevipalpis* diet with folate makes this tsetse species significantly more permissive to trypanosome establishment [31], highlighting trypanosome reliance on symbiont generated nutrients for successful vector infection. Here, metabolic properties of both tsetse and its bacterial symbionts may play key roles in trypanosomal nutrition and may signal parasite developmental cues that may ultimately impact the outcome of an infection. Genes in this category include those directly involved in vitamin and cofactor biosynthesis, such as those in the COG category of “Coenzyme transport and metabolism”. Furthermore, a higher expression of *Sodalis* genes involved in chitin metabolism, such as the high expression of exochitinase and chitin-binding protein observed in the *G. morsitans* isolates, may have direct effects on trypanosome-tsetse interactions facilitating parasite establishment via competitive interference with lectins, while compromising the physical robustness of the PM which is primarily constituted of peritrophins and chitin. Lectin abundance within the midgut increases with tsetse age [110], therefore the breakdown of chitin by *Sodalis* would be less damaging towards anti-trypanosomal efforts as tsetse age [118]. Furthermore, chitin-binding proteins are thought to work in conjunction with exochitinases facilitating cell adhesion to cellular targets (reviewed in [119]), and orthologs have been implicated as virulence factors in various bacterial infections (reviewed in [120]).

The transcriptomic profile of *Wigglesworthia* flagellar genes represents two different stages of flagella synthesis between the species isolates. Flagella facilitate fine-tune responses to environmental stimuli by bacteria [121] and their regulation and synthesis is metabolically expensive, stressing the importance of global and master regulators genes to control their expression. The master regulator *flhDC* is transcribed into a single mRNA and translated into two distinct proteins FlhD and FlhC which assemble into the hexameric complex FlhD_4_C_2_ [122]. This complex attaches to promoter regions upstream of class II flagellar genes [51] facilitating ribosomal recruitment and transcription. Given the temporal regulation of this cascade (reviewed in [123, 124]), the stability of FlhD_4_C_2_ is under tight control [125] with even the degradation of the *flhDC* mRNA controlled by global regulators such as CsrA [126, 127]. However, as *csrA* orthologs are absent in *Wigglesworthia* genomes, the regulation of master control genes such as *flhDC* and the potential role tsetse may play towards mediating flagellar control deserves further investigation. With this understanding, our results which show contrasting levels of *flhDC* transcription by *Wigglesworthia* may be due to measuring gene expression at a single time point. Despite the great advantage of looking at all the genes present in a pathway simultaneously, RNASeq lacks the temporal resolution necessary to reflect the dynamic nature of regulatory processes. This temporal regulation may explain our results, as for example, the *G. morsitans* isolates expression pattern may represent a stage where the *flhDC* mRNA is being highly transcribed, but it has yet to be translated to go on and exert its activation on class II gene transcription. This would account for a comparatively high expression of *flhDC* operon while the class II genes are still at low levels. Conversely, the pattern observed in *G. brevipalpis* may reflect a stage further in the flagellar synthesis cascade where the *flhDC* mRNA was already transcribed and mostly degraded, while the corresponding protein complex is activating (or has already) the transcription of downstream class II flagellar genes. Further studies following the mRNA stability and protein levels of *flhDC* across tsetse development, specifically encompassing the late pupae to early adult transition, may clarify the diverging regulation dynamics behind these expression patterns with likely implications towards acquisition of the obligate *Wigglesworthia* critical for tsetse developmental biology. Inhibiting the vertical transmission of *Wigglesworthia* renders female progeny sterile and may serve as a novel angle for next generation pesticides with sole specificity to the tsetse fly.

The low number of read counts arising from *Sodalis* is not surprising given that the abundance of 16S rRNA sequences belonging to *Sodalis* is relatively low in *G. morsitans* [12, 128]. The small proportion of differentially expressed genes in *Sodalis*, in comparison to *Wigglesworthia,* may reflect differences in symbiont acquisition times where the ancient *Wigglesworthia* is particularly fine-tuned to its host species due to their extensive co-evolutionary history. In contrast, *Sodalis* is in an incipient stage of co-diversification [129, 130]. Two other notable features of the *Sodalis* transcriptome include the significantly greater proportion of highly expressed genes (> 100 TPM) within the *G. morsitans* midgut isolates and the wide range of *Sodalis* genes expressed within *G. brevipalpis* individuals.

### Tsetse fly fitness requires a balanced interaction with its microbiome and the environment

Invertebrates depend on innate immunity to mediate responses to microorganisms. These interactions may antagonize an infection, neutralize a pathogen, or even establish permissive (i.e. tolerance) conditions, thus promoting beneficial symbioses [78]. Beneficial symbionts have been found to orchestrate a coordinated development of the host immune system that simultaneously allows persistence while still enabling protection from pathogens. For example, *Burkholderia* symbionts differentially suppress host immunity to allow persistence [131] while playing a critical role in the proper functioning of the immune system in the bean bug *Riptortus pedestris* [132]. Similarly, *Wigglesworthia* is essential for the development of the immune system in tsetse [33, 34], given that larvae reared in the absence of this symbiont exhibit compromised humoral and cellular immune responses to pathogens as adults [34, 35].

The transcriptomic profiles of immunity related genes we observe in tsetse midguts and bacteriomes are consistent with an attenuated response to symbionts, rather than with “on” or “off” states, as for example regarding humoral immunity. The Toll pathway generally responds to challenge by Gram-positive bacteria and fungi, while the IMD pathway is dedicated towards the surveillance and protection against Gram-negative bacteria and fungi [89]. Our bacteriome libraries indicate that the Toll pathway is inactive in tenerals, not surprising, given that the sequencing libraries were generated from laboratory reared teneral flies lacking exposure to exogenous bacterial challenges. However, the Imd pathway is active within tenerals, as may be expected given that the tsetse microbiota is dominated by the Gram-negative bacteria, *Sodalis* and *Wigglesworthia*. However, these natural infections represent a physiological challenge for the tsetse, particularly due to the essential nature of its association with *Wigglesworthia*. Tsetse appear to have evolved mechanisms to circumvent this challenge by differential activation of the pathway within bacteriomes versus midguts and through the targeted expression of genes such as *pgrp-lb*, which when translated scavenges peptidoglycan released during *Wiggleworthia* cell division, thus preventing the activation of the hostile IMD pathway [36] which would prove detrimental towards *Wigglesworthia*.

Interestingly, several observations support an enhanced readiness in *G. brevipalpis* to deter trypanosome infections as tenerals. Previous transcriptome analyses in tsetse have found that activators of both Imd and Toll pathways are present in the fat body [133] and are necessary to counteract trypanosome infections and decrease their density [134]. The *imd* gene is highly expressed in *G. brevipalpis* midguts along with a concomitant high expression of the antimicrobial peptide cecropin. The cecropin peptide has potent killing activity against the American trypanosome *Trypanosoma cruzi*, [92, 93], so similar deleterious effects against African trypanosomes may thus be hypothesized. Additionally, genes for enzymes that initiate the melanization cascade (i.e. MP1-Sp7) and their corresponding inhibitors (Serpin 27A) both have a higher expression in *G. brevipalpis*, which may enable a faster deployment should a trypanosome infection occur, as phenoloxidases have been implicated in response to trypanosome challenges in other insects such as triatomines [135] and bumble bees [136]). Consistent with this rationale, a pre-activation of immunity via artificial bacterial challenge, enables tsetse to respond more efficiently to a trypanosome challenge [74]. It is plausible that tsetse species provide microenvironments with different degrees of biological hostility toward trypanosomes, which would translate into distinctions in vector competence.

The differential expression observed in the set of genes arising from tsetse and its symbionts becomes even more relevant, as these occur at the teneral state. It seems possible that following adult metamorphosis, *G. brevipalpis* is comparatively better suited than *G. morsitans* to counter trypanosome infections. These gene expression patterns warrant a deeper investigation. For example, selective knockdown of genes or their activity, through RNA interference or chemically mediated inhibition, followed by trypanosome challenge would provide avenues targeted at assessing the contribution of identified genes towards vector competence.

## Conclusions

The tsetse fly, similar to other animals, has to balance protection against pathogens with the biological integration of its essential microbiome. Our results indicate that this equilibrium may be, at least partially, achieved via a comparative downregulation of immunity in the compartments that harbor essential symbionts (i.e. bacteriomes in tsetse) relative to a direct route for pathogen entry, such as the digestive tract. Here, we show that bacterial symbionts exhibit transcriptomic profiles that reflect the duration of their respective host co-evolutionary histories, with a high percentage of DEGs in the ancient *Wigglesworthia* and a significantly lower proportion of DEGs in the more recent *Sodalis* when comparing tsetse species isolates. Furthermore, observed differences in the metatranscriptomes of the two tsetse species considered, such as the putatively higher deployment of antimicrobial peptide cecropin by *G. brevipalpis*, or higher transcription of enzymes with predicted chitinolytic activity in the *Sodalis* isolate from *G. morsitans*, offer insight into mechanisms that may predispose tsetse species to trypanosome establishment.

## Methods

### Insect rearing

*Glossina morsitans morsitans* pupae were provided by the Institute of Zoology, Slovak Academy of Sciences (Bratislava, Slovakia), and *Glossina brevipalpis* pupae were supplied by the Joint FAO/IAEA Division of Nuclear Techniques in Food and Agriculture (Vienna, Austria). Pupae were maintained in the Department of Biology insectary at West Virginia University at 24 ± 1 °C with 55% relative humidity on a 12-h light/12-h dark schedule until adult eclosion. Teneral flies (newly emerged adults prior to blood meal acquisition) were collected < 24 h post emergence from pupae and sorted by sex. Flies included in this study were all trypanosome free.

### Dissections and RNA extraction

Bacteriomes and intestinal tracts (flanked by the bacteriome and the Malpighian tubules) were microscopically dissected and placed in RNA*later* (Invitrogen, Carlsbad, CA) at − 20 °C. The bacteriomes or intestinal tracts of 20 teneral tsetse of each sex and species were pooled for one biological sample, resulting in a total of 18 biological samples used in our analyses. Bacteriomes and guts were homogenized and total RNA was extracted using a MasterPure RNA purification kit (Epicentre, Madison, WI) according to the manufacturer’s protocol for tissue samples. DNA was removed from the RNA samples using a Turbo DNA-free kit (Ambion, Austin, TX) following the rigorous DNase treatment option. The RNA concentration was measured using a Qubit fluorometer (Thermo Fisher Scientific, Waltham, MA) with an Agilent 2000 Bioanalyzer RNA Nano chip used to validate RNA sample quality and integrity.

### mRNA library preparation, sequencing, and genome alignment

Library preparation was performed at the WVU Genomics Core Facility by using 1 μg of total RNA and Ribo-Zero Gold Epidemiology kit (Illumina, San Diego, CA) following the manufacturers recommended protocol. Following cDNA synthesis, libraries were quantified via Qubit fluorometer with high sensitivity DNA reagent and run on an Agilent high sensitivity DNA chip to determine average library size. The libraries were pooled in equimolar amounts and sequenced using the Illumina HiSeq 1500 platform (2 by 51 bp) at Marshall University. Following sequencing, raw reads were postprocessed to remove Illumina adapter and barcode sequences.

FastQC (http://www.bioinformatics.bbsrc.ac.uk/projects/fastqc) analysis, as implemented in MultiQC [137], was performed on the RNA-Seq data sets to validate read quality. Reads were mapped to the *Glossina morsitans* (GCA_001077435.1)*, Glossina brevipalpis* (GCA_000671755.1)*, Wigglesworthia* (GCA_000008885.1) and *Sodalis glossinidius* (GCA_000010085.1) genomes using Salmon [29]. Gene transcription level was converted to Transcripts per Million (TPM) fragments mapped and used for statistical comparison of expression levels between libraries [138]. Statistically significant differences were accepted at *p* < 0.05 with adjusted *p-*values for multiple testing.

### Principal Component Analysis (PCA)

PCAs comparing gene TPM values were performed with the prcomp package (version 3.6.3) in the R software suite. PCA excluded genes that lacked expression across all libraries. PCA plots were visualized using the R package ggbiplot.

### Differential expression analyses

IDEAmex [52], which implements the R packages DESeq2 [139], edgeR [140], limma [141] and NOISeq [142], with an adjusted *p*-value cut-off of 0.05 was used to compare gene expression profiles between *Wigglesworthia* isolates, between *Sodalis* isolates and between tsetse species. To compare *Wigglesworthia* gene expression between sexes within a species, the mapped read counts were initially used as input for DESeq. Additionally, EggNOG mapper [143] was used to assign Clusters of Orthologous Groups (COG, [49]) to categorically summarize annotation data into specific biological categories that were enriched within specific libraries.

### Validation of differential expression through qRT-PCR

A subset of genes identified to be differentially expressed between libraries was verified via quantitative reverse transcription PCR (qRT-PCR). For the validation of the differential expression of *Wigglesworthia* genes*,* biological samples that consisted of six individual bacteriomes from either tsetse sex were collected in RNAlater (ThermoFisher Scientific, Waltham, MA) following manufacturer’s protocol. Total RNA was isolated using MasterPure™ RNA (Epicentre, Madison, WI) and treated with TURBO™ DNase (ThermoFisher Scientific, Waltham, MA) following the rigorous treatment protocol to remove contaminant DNA. Linearized plasmid standards used for the quantification of gene copy number were made for respective genes using the pGEM®-T Vector Systems (Promega, Madison, WI) according to manufacturer instructions. Table S1 includes a list of primers used for cloning and qPCR amplification. First-strand cDNA synthesis was performed with SuperScript™ II Reverse Transcriptase (ThermoFisher Scientific, Waltham, MA). Second-strand cDNA synthesis was performed with a CFX96 Real-Time PCR Detection System (Bio-Rad, Hercules, CA) using the SsoFast™ PCR cocktail (Bio-Rad) and following the conditions used in Additional file 10: Table S9. Three technical replicates were performed for each biological sample and averages obtained. The relative gene expression was determined for selected genes using the 2^-ΔΔCt^ method [144].

### Immunity

Putative orthologs of *D. melanogaster* immunity genes [80] in tsetse were identified by using FlyBase (https://flybase.org). Orthologs between *G. morsitans* and *G. brevipalpis* were identified through VectorBase (https://vectorbase.org). The list of immune-related orthologs was validated and placed into pathways according to [81].

### Graphs and statistical analyses

Heatmaps were generated with the ‘heatmap.2 function’ in the gplots R package with clustering by species/tissue type based on the distinct expression patterns between isolates. GraphPad Prism was used for statistical analyses with *p*-values < 0.05 considered statistically significant.

## Supplementary Information

**Additional file 1: Fig. S1.** Mean quality scores by position of the reads. **Fig. S2.** Read count per library. **Fig. S3.** Comparison of total reads and mapped reads between tsetse species libraries. **Fig. S4.** Within species comparison of highly expressed *Wigglesworthia* genes among two tsetse species isolates.

**Additional file 2: Table S1.** Differentially expressed *Wigglesworthia* genes between female and male *G. morsitans* isolates.

**Additional file 3: Table S2.** Upregulation and downregulation of differentially expressed genes between *Wigglesworthia* isolates.

**Additional file 4: Table S3.** Highly expressed *Sodalis* genes.

**Additional file 5: Table S4.** *p*-values of differentially expressed genes between *Sodalis* isolates.

**Additional file 6: Table S5.** *p*-values of differentially expressed genes between *G. brevipalpis* and *G. morsitans* in bacteriome libraries.

**Additional file 7: Table S6.** *p*-values of differentially expressed genes between *G. brevipalpis* and *G. morsitans* in midgut libraries.

**Additional file 8: Table S7.** *p*-values of differentially expressed genes between *G. brevipalpis* bacteriomes and midgut libraries.

**Additional file 9: Table S8.** *p*-values of differentially expressed genes between *G. morsitans* bacteriomes and midgut libraries.

**Additional file 10: Table S9.** Primers used for the validation of *Wigglesworthia* differential expression though qRT-PCR.

## Data Availability

Raw reads are publicly available in the Short Reads Archive (SRA) of the National Center for Biotechnology Information (Bio-project PRJNA668823; https://www.ncbi.nlm.nih.gov/bioproject/PRJNA668823 entitled Metatranscriptome of teneral tsetse flies of varying vector competence).

## Abbreviations

AMPs: Antimicrobial peptides
COGs: Clusters of orthologous groups of proteins
DEGs: Differentially expressed genes
PCA: Principal component analysis
PM: Peritrophic matrix
PPO: Prophenoloxidase
ROS: Reactive oxygen species
TPM: Transcripts per Million
