# Supplementary material for: The holobiont transcriptome of teneral tsetse fly species of varying vector competence
Source: BMC Genomics. 2021 May 31;22:400. doi: 10.1186/s12864-021-07729-5 (PMC8166097; doi:10.1186/s12864-021-07729-5)
Supplement: Supplementary file 1 — Additional file 1: Fig. S1. Mean quality scores by position of the reads. Fig. S2. Read count per library. Fig. S3. Comparison of total reads and mapped reads between tsetse species libraries. Fig. S4. Within species comparison of highly expressed Wigglesworthia genes among two tsetse species isolates. [file 12864_2021_7729_MOESM1_ESM.pdf]

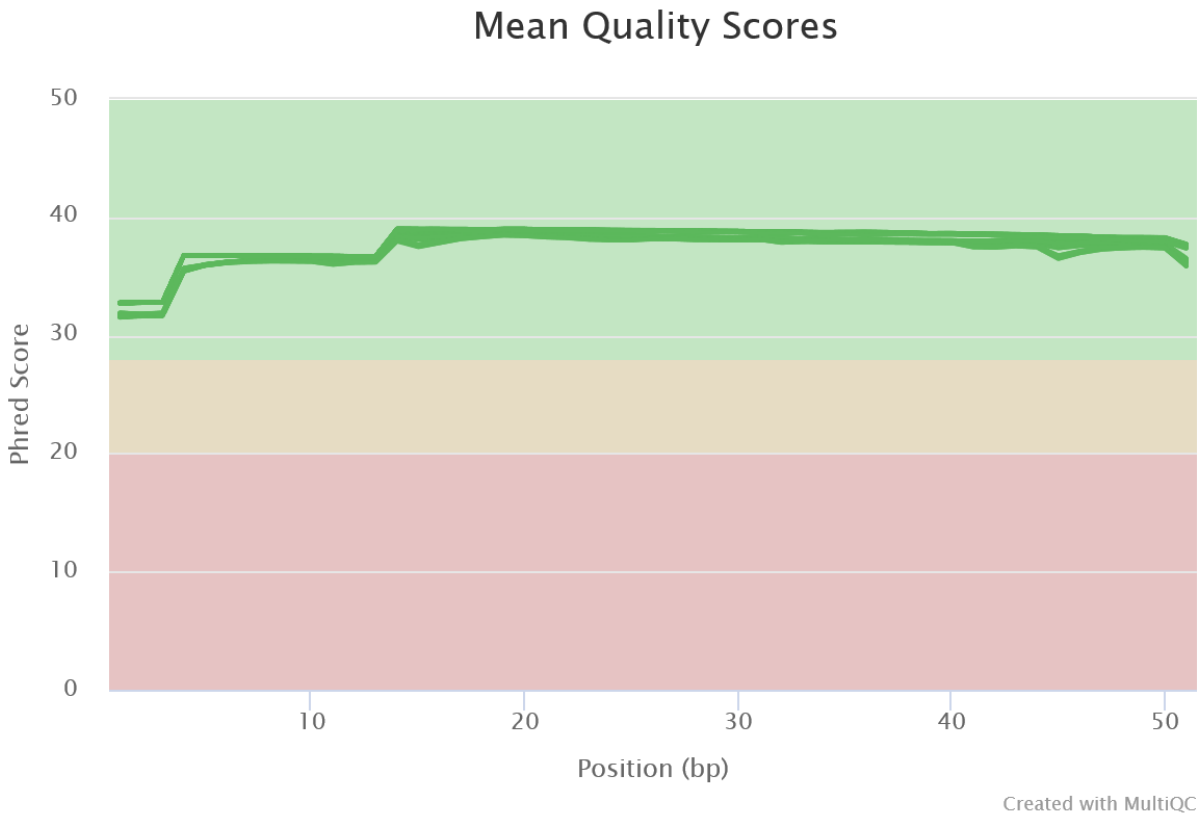

Figure S1. Mean quality scores by position of the reads. The quality score is displayed on the y-axis, higher scores represent better qualities. The background classifies the scores into very good (in green), reasonable (in orange), and poor (in red) quality. All reads in this study were of very good quality and did not require trimming.

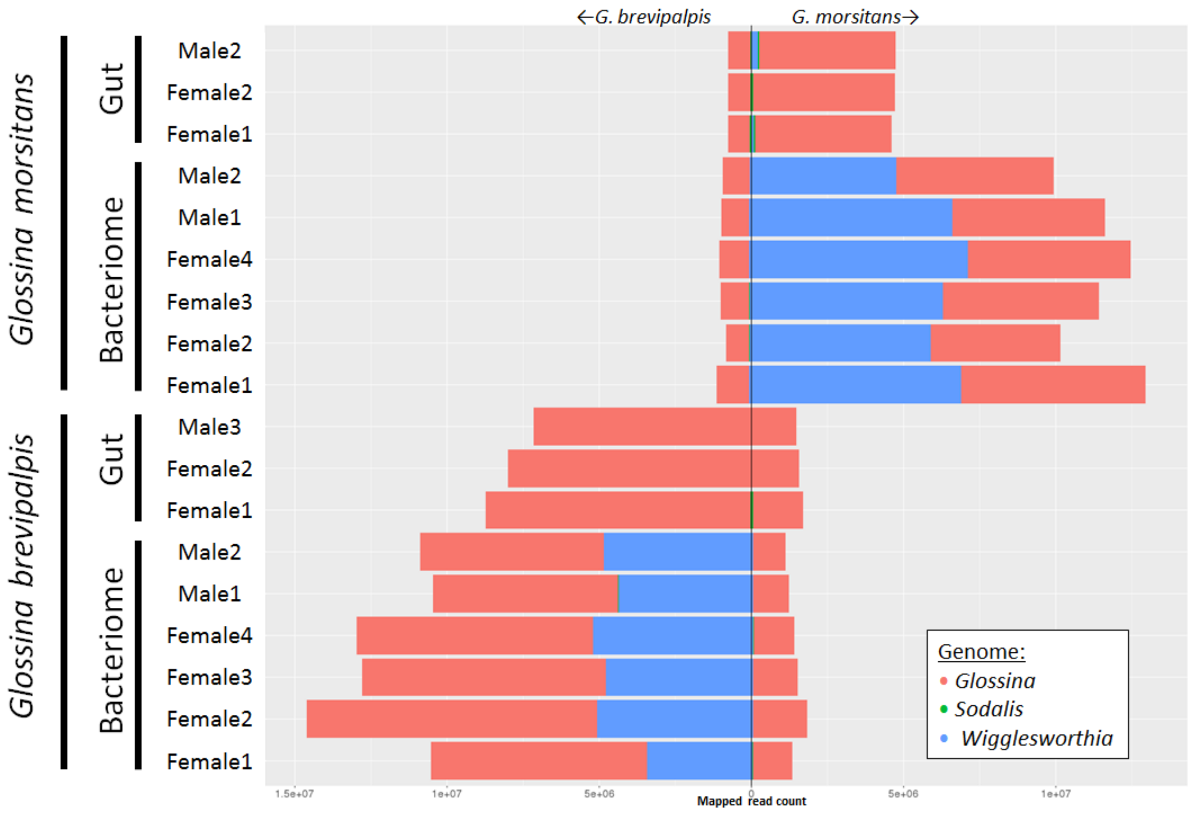

Figure S2. Read count per library. Bar lengths represent the read count with colors indicating the genome to which the reads mapped. Reads were mapped to a construct containing either *G. brevipalpis-W. glossinidia brevipalpis-S. glossinidius* (left) or *G. morsitans-W. glossinidia morsitans-S. glossinidius* (right). The same *S. glossinidius* genome was used in both constructs, as only one *Sodalis* genome from tsetse was available at the time of analysis.

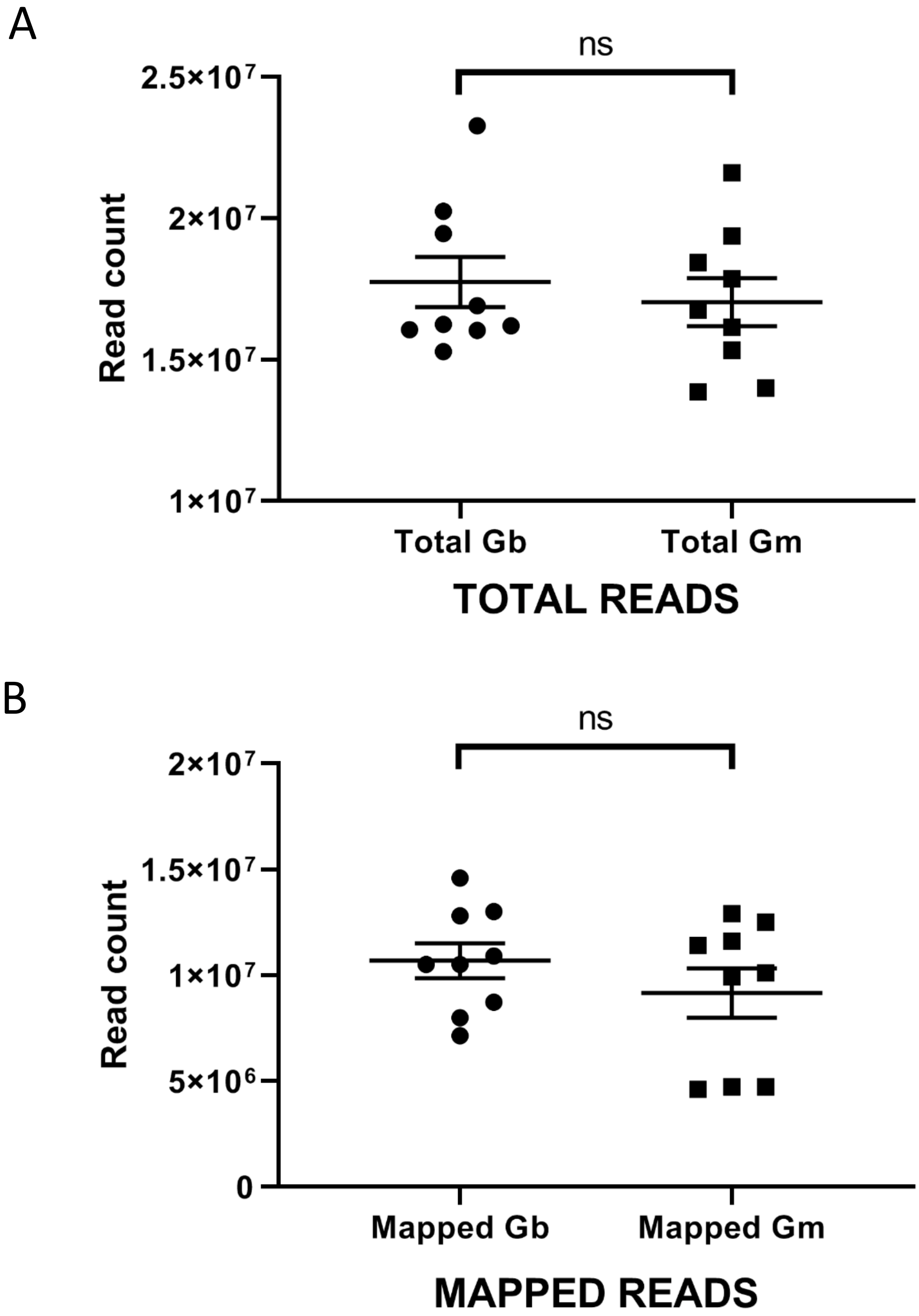

Figure S3. Comparison of total reads and mapped reads between tsetse species libraries. A. Total reads mean ± SEM, Unpaired Student's *t*-test. B. Mapped reads mean ± SEM, Mann-Whitney test.

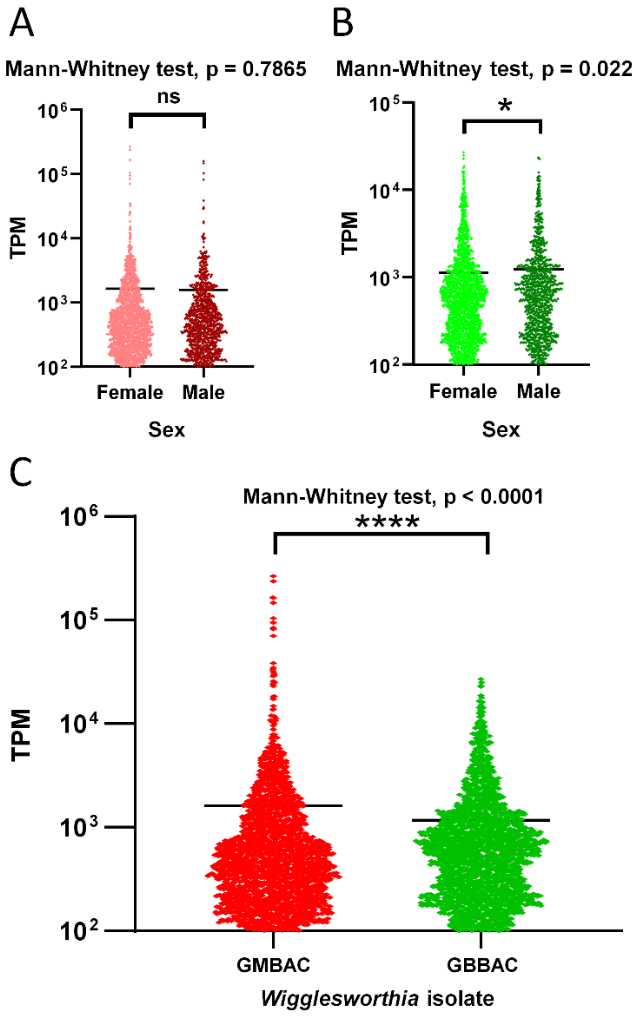

Figure S4. Within species comparison of highly expressed *Wigglesworthia* genes among two tsetse species isolates. Highly expressed genes were defined as loci with expression levels of $\geq$ 100 TPM. A. Comparison of mean levels of highly expressed genes in *Wigglesworthia* expression between sexes for *G. morsitans* isolates (Mean TPM $\pm$ SEM; Mann-Whitney test). B. Comparison of mean levels of highly expressed genes in *Wigglesworthia* expression between sexes for *G. brevipalpis* isolates (Mean TPM $\pm$ SEM; Mann-Whitney test). C. Comparison of mean *Wigglesworthia* expression of highly expressed genes within bacteriome libraries between the tsetse species (Mean TPM $\pm$ SEM; Mann-Whitney test).
